# Research trends and hotspots in colorectal cancer screening: Bibliometric and visualization analysis (2015–2024)

**DOI:** 10.1097/MD.0000000000051198

**Published:** 2026-10-16

**Authors:** Jinkai Zhou, He Zhou, Jiayu Xu, Maohua Wang, Lei Liu, Xiaolong Tang

**Affiliations:** a The Second Department of Gastrointestinal Surgery, Affiliated Hospital of North Sichuan Medical College, Nanchong, China; b Institute of Hepatobiliary, Pancreatic and Intestinal Disease, North Sichuan Medical College, Nanchong, China; c School of Clinical Medicine, North Sichuan Medical College, Nanchong, Sichuan, China.

**Keywords:** bibliometric analysis, colon cancer, colonoscopy, colorectal cancer, colorectal cancer screening, CRC, screening

## Abstract

**Background:**

Colorectal cancer (CRC), is a highly prevalent malignant tumor worldwide, and early screening is key to improving its prognosis. Currently, CRC screening research is growing exponentially, but its core research hotspots, evolutionary trends, and interdisciplinary features are not clear. In this study, we used bibliometric analysis to analyze the distribution of literature, research hotspots, and cutting-edge developments in the field of CRC screening worldwide, and to provide a scientific basis for optimizing screening strategies.

**Methods:**

Publications on CRC screening from 2015 to 2024 were retrieved from the Web of Science Core Collection. VOSviewer (v1.6.20), RStudio (v4.4.1), and CiteSpace (v6.4.2) were used for bibliometric and visual analyses. The main analyses included publication trends, countries, authors, journals, references, and keywords.

**Result:**

A total of 3245 publications were included in the analysis. Global publications on CRC screening showed a steady upward trend over the past decade, with an annual growth rate of 2.17%. The United States contributed the highest number of publications, and the University of North Carolina ranked as the most productive institution. GASTROENTEROLOGY and GUT were the most frequently cited journals in this field. Lansdorp‑Vogelaar I was among the most prolific authors. The most cited article was “CRC screening for average-risk adults: 2018 guideline update from the American Cancer Society.” Keyword analysis showed that the research hotspots in this field included colonoscopy, biomarker, adenoma detection rate, artificial intelligence, etc.

**Conclusion:**

Current research in CRC screening focuses on traditional screening methods, population-related factors, and emerging technologies, particularly artificial intelligence. Stronger international collaboration and interdisciplinary studies are needed to advance the field.

## 1. Introduction

Colorectal cancer (CRC) ranks third in incidence and second in lethality among malignant tumors globally. In 2022, there were more than 1.9 million new cases of CRC and about 904,000 deaths globally.^[1]^ Most patients are diagnosed at advanced stages because early CRC often has no obvious symptoms.^[2]^ CRC usually develops slowly from precancerous lesions, a characteristic that renders it one of the few cancers whose prognosis can be improved by population screening. Randomized controlled trials have confirmed that screening reduces both the incidence and mortality of CRC.^[3,4]^ Additionally, both the U.S. Preventive Services Task Force^[5]^ and the U.S. Multi-Society Task Force^[6]^ on CRC strongly recommend screening in asymptomatic populations. These studies demonstrate that screening plays a pivotal role in the control of CRC.

Research on CRC screening has shown steady growth over the past decade.^[7,8]^ It is challenging for researchers to keep track of overall trends, major contributors, and evolving topics in the field. Traditional reviews may not objectively reflect the broader research landscape because they rely on the selective inclusion of studies. Bibliometric analysis uses quantitative methods and visualization tools to systematically map publication outputs, collaboration networks, and knowledge structures. This approach can objectively identify influential studies, emerging topics, and future research directions. Currently, some bibliometric studies have already examined early cancer screening.^[9–11]^ Most of them focused on specific biomarker detection, such as circulating tumor DNA or microRNA, shorter time coverage, and limited their scope to certain countries or regions.^[12]^ A comprehensive global bibliometric analysis of CRC screening over the past decade remains relatively scarce.

In this research, we employed leading bibliometric and science mapping tools to analyze publications on CRC screening from the Web of Science Core Collection. The aim was to identify key publications, influential contributors, and emerging hotspots while visualizing the evolution of research trends, thereby assisting researchers in tracking research frontiers and inspiring new research directions.

## 2. Materials and methods

### 2.1. Data collection

The Web of Science Core Collection is used as the source of literature, and the search strategy is TS= (“CRC screening” OR “colon cancer screening” OR “rectal cancer screening” OR “colorectal carcinoma screening” OR “rectal carcinoma screening” OR “colon carcinoma screening” OR “colorectal neoplasm screening” OR “rectal neoplasm screening” OR “colon neoplasm screening”). The literature search was performed on April 14, 2025, with the time frame from January 1, 2015 to December 31, 2024. The language selected is “English,” the type of literature is “Article” and “Review Article.” Exclude duplicate publications and literature unrelated to the topic. The final search result is 3245 articles. The year of final publication is used in this study. The detailed search strategy and literature screening process are illustrated in Figure 1.

**Figure 1.**
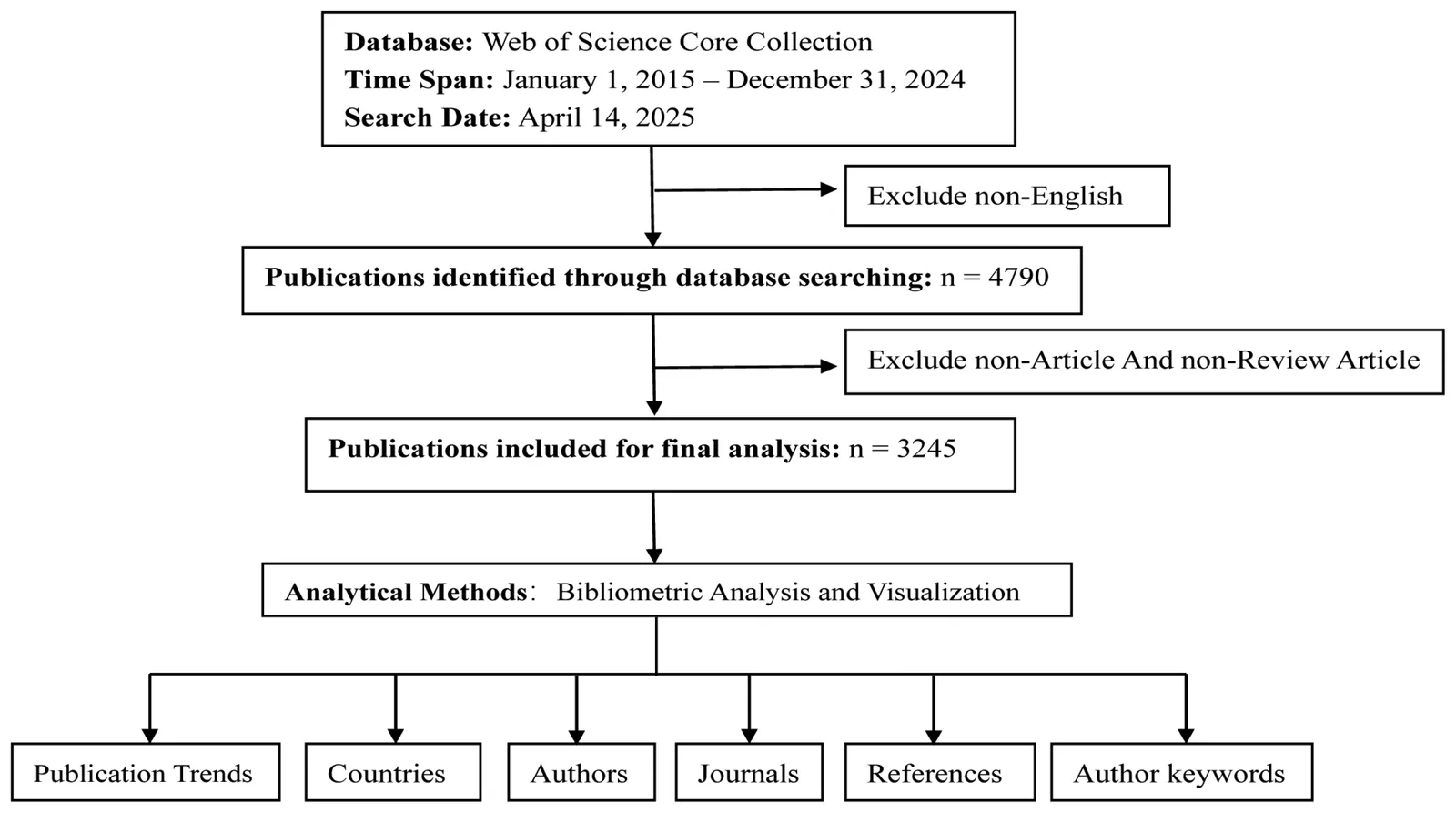
Retrieval flowchart.

### 2.2. Data analysis

VOSviewer is a Java-based tool for constructing and visualizing scientific networks, including co-citation, collaboration, and keyword co-occurrence maps.^[13]^ The bibliometrix R package integrates a comprehensive suite of statistical and visualization functions for bibliometric analysis, enabling in‑depth exploration of literature datasets to detect emerging trends, thematic evolution, and intellectual landscapes.^[14]^ CiteSpace is another Java-based application designed to analyze collaborative relationships, research priorities, and emerging trends in a given field.^[15]^ Using these tools, we analyzed publication counts by year, as well as information on titles, countries/regions, institutions, authors, journals, average citations, keywords, and references.

Before analysis, duplicates were removed using the R-based bibliometrix package based on DOI matching, leaving 3245 records. VOSviewer was used to analyze collaborations among countries, institutions, authors, co-cited authors, journals, and keywords, with a minimum threshold of 25 for all parameters under the full counting method. In the maps, node size shows publication volume, color shows cluster membership, and line thickness shows collaboration strength. CiteSpace was used for keyword clustering and burst detection, with a 1‑year time slice throughout. The g‑index (*k* = 10) was applied to select the most influential nodes in each time slice. LLR algorithm for clustering, and gamma = 0.5 for burst detection. Node size reflects term frequency. For clustering, *Q* > 0.3 means significant clustering, and *S* > 0.7 means high reliability. This study is a bibliometric analysis based on publicly available published literature. Therefore, ethical approval and informed consent were not required.

## 3. Results

### 3.1. Data overview in publication outputs

A total of 3245 publications were retrieved from 2015 to 2024, with an annual growth rate of 2.17%. Among these, 2863 were original articles and 382 were reviews, involving 14,770 authors, with an average of 19.99 citations per paper and 7.61 coauthors per document (Table 1). The annual publication and total cumulative citation trends over the past decade are shown in Figure 2. Overall, the annual number of publications showed a fluctuating upward trend, reflecting growing research interest and scientific exploration in the field of CRC screening, and indicating its continued activity and development potential. Annual citations decreased from 39 in 2015 to 2 in 2024, which may be related to the fact that earlier papers had more time to accumulate citations.

**Table 1:** Data overview of papers related to colorectal cancer screening.

Description	Results
Main information about data	
Timespan	2015:2024
Sources (Journals, Books, etc)	578
Documents	3245
Annual growth rate %	2.17
Document average age	5.1
Average citations per doc	19.99
References	61502
Document contents	
Keywords Plus (ID)	3349
Author’s Keywords (DE)	4242
Authors	
Authors	14770
Authors of single-authored docs	42
Authors collaboration	
Single-authored docs	44
Co-Authors per Doc	7.61
International co-authorships %	17.23
Document types	
Article	2841
Article; book chapter	1
Article; data paper	1
Article; proceedings paper	20
Review	379
Review; book chapter	3

**Figure 2.**
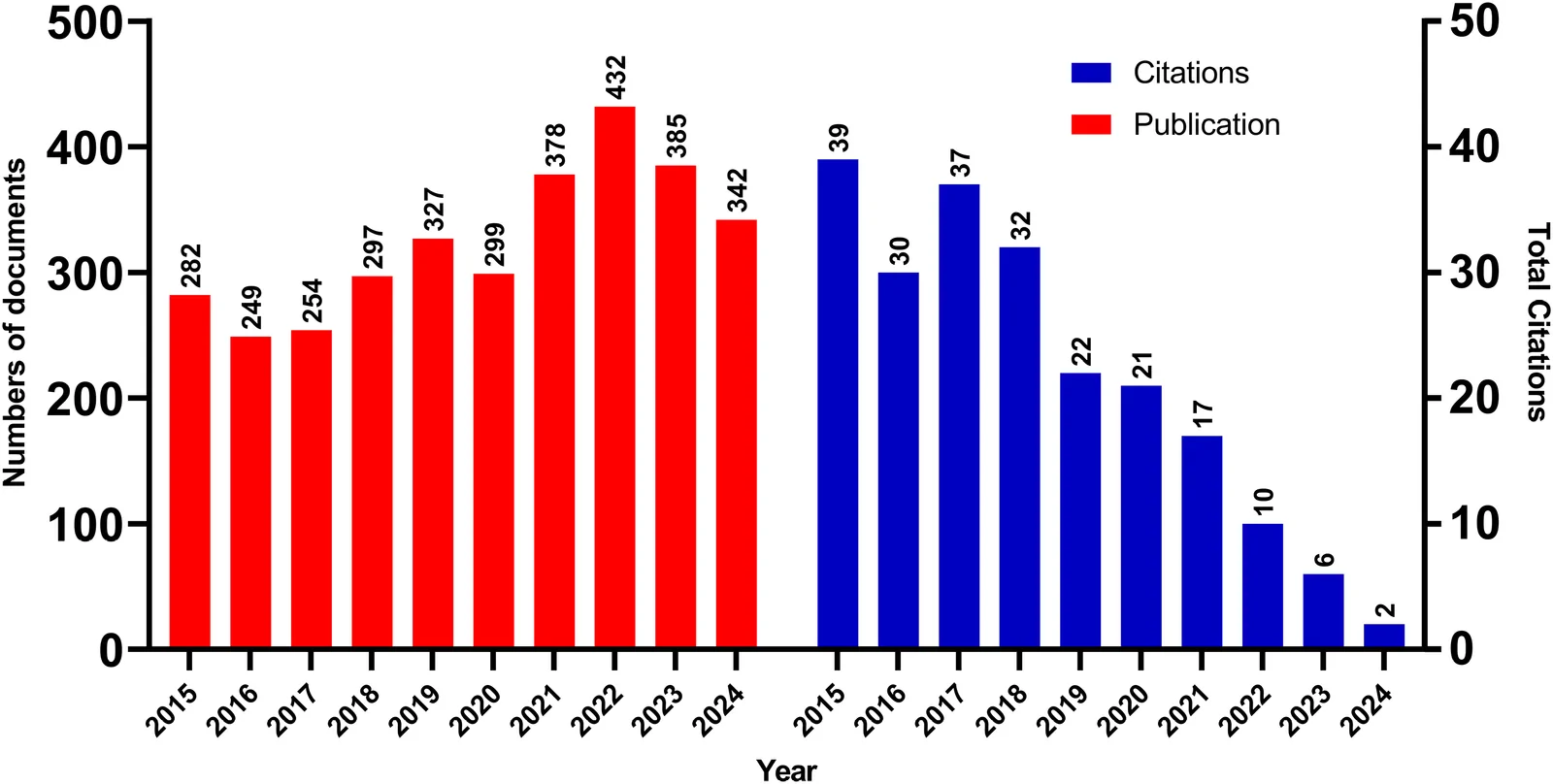
Annual publication counts and total cumulative citations (retrieved on April 14, 2025). The citations for each year indicate the total number of citations accumulated by all publications from that year up to the data retrieval date.

### 3.2. Distribution of countries/regions and institutions

The bibliometric analysis of countries and institutions allows us to understand the research contribution of each country and institution in the field of CRC screening in order to better navigate and participate in the field. In total, 65 countries contributed to publications in this field. The United States, China, the Netherlands, Canada, and Spain were the top five most productive countries, with 1423, 259, 159, 140, and 136 publications, respectively, accounting for 65.23% of all included papers (Fig. 3A). Additionally, the United States, Netherlands, and China ranked highest in total citations. Regarding international collaboration, the Netherlands and Italy had the highest proportions of internationally coauthored papers, at 34.6% and 32.5%, respectively (Table 2). In contrast, although the United States contributed the largest absolute number of multi-country collaboration publications (105; Fig. 3B), its corresponding ratio was relatively low at 7.4% (Table 2). The Netherlands had the highest average citations per paper, followed by Italy and the USA, suggesting relatively high per‑article impact for these countries (Fig. 3C). However, it should be noted that recent publications have had less time to accumulate citations. The United States collaborated most closely with Canada, China, and Spain, while China’s primary collaborative partners were Japan, the United Kingdom, and Australia (Figure 4A–B). The United States also served as a central hub in the collaboration network, with strong ties to multiple countries and regions (Fig. 5A). A total of 2044 institutions contributed to publications in this field, predominantly from the United States, Canada, and the Netherlands (Supplementary Table 1, Supplemental Digital Content 1). Among these, the University of North Carolina ranked first with 488 publications. The institutional collaboration network (Fig. 5B) revealed close cooperative relationships among major research institutions.

**Table 2:** Top 10 countries with the most published articles.

Country	Articles	Articles %	SCP	MCP	MCP %	TC	TC/Articles
USA	1423	43.9	1318	105	7.4	31303	22
China	259	8	230	29	11.2	4038	15.59
Netherlands	159	4.9	104	55	34.6	5162	32.47
Canada	140	4.3	108	32	22.9	2903	20.74
Spain	136	4.2	114	22	16.2	2222	16.34
United Kingdom	135	4.2	104	31	23	2583	19.13
Italy	117	3.6	79	38	32.5	2988	25.54
Germany	85	2.6	67	18	21.2	1654	19.46
Denmark	78	2.4	54	24	30.8	1074	13.77
France	77	2.4	58	19	24.7	1629	21.16

MCP = multi-country collaboration publications, SCP = single country publications, TC = total citations.

**Figure 3.**
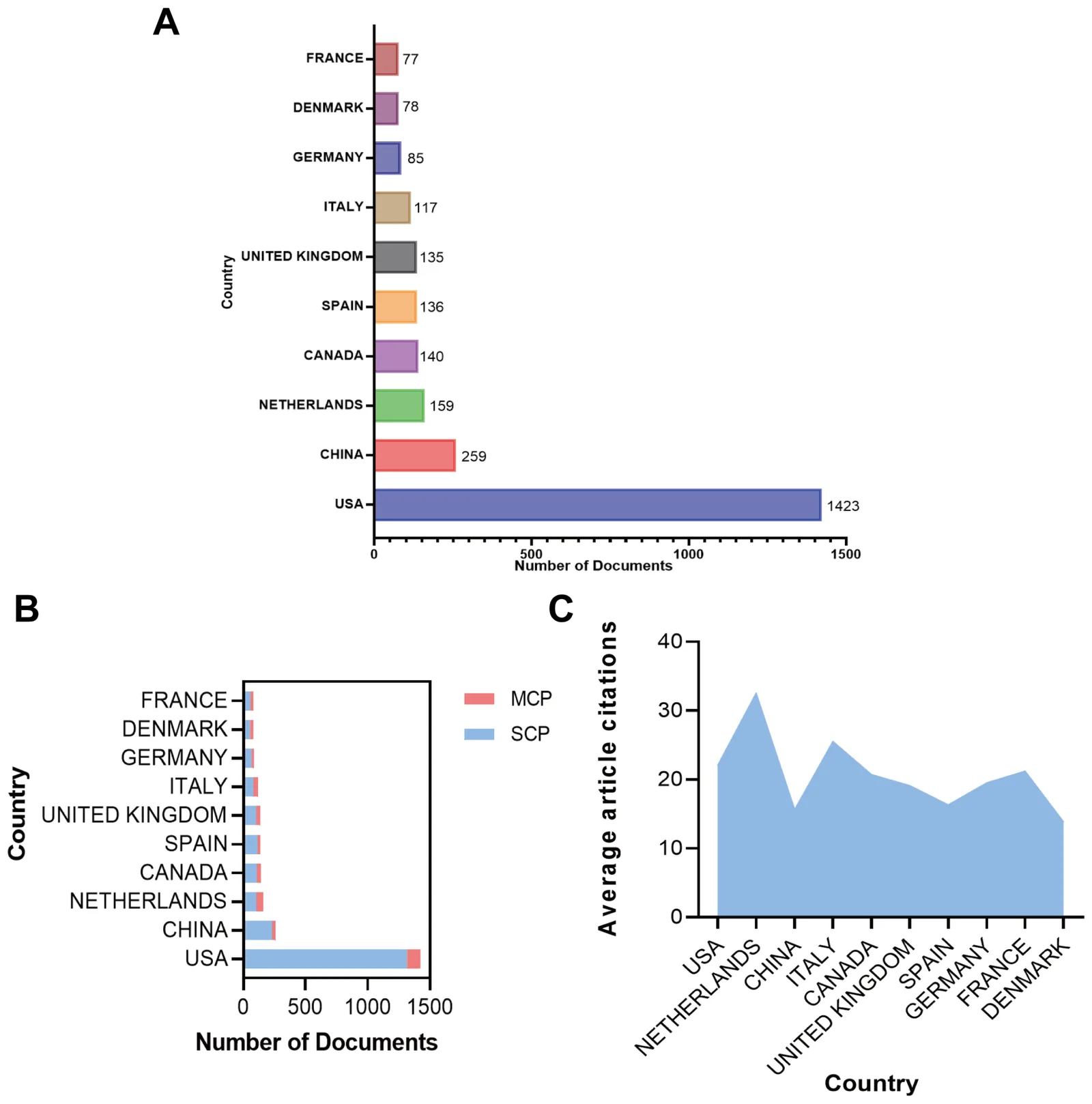
Paper publication status in various countries. (A) Number of publications from the top 10 countries. (B) Patterns of international collaboration in paper publication: Single Country Publications (SCP); Multi-country Collaboration Publications (MCP). (C) Average article citations of each country.

**Figure 4.**
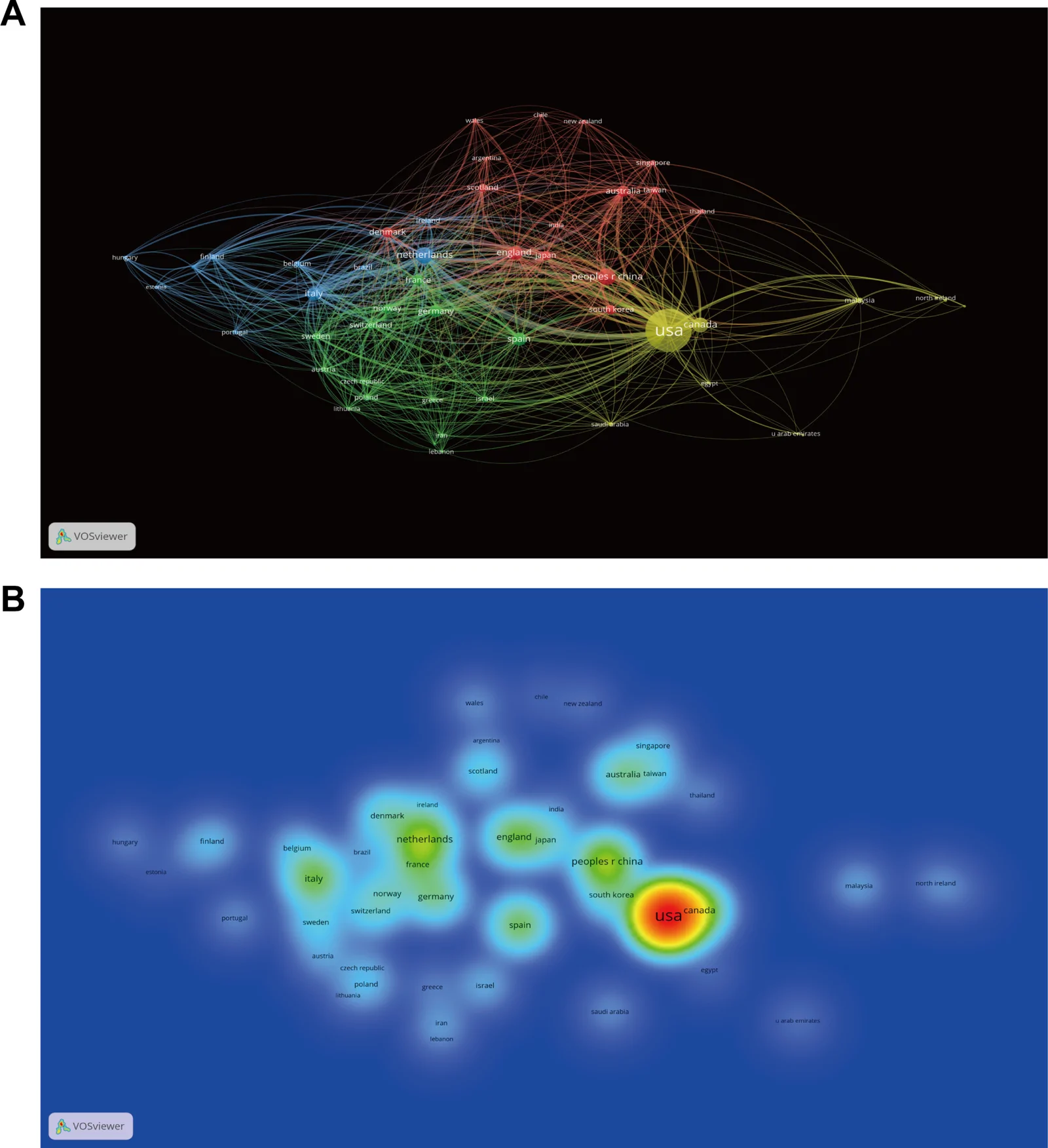
The visualization of cooperation between countries by VOS viewer. (A) International cooperation network diagram. The same-colored logo indicates the cluster group of countries, the thickness of the line reflects the closeness of cooperation, and the node’s size corresponds to the number of papers published by each country. (B) Heat map of cooperation analysis between countries. Each label represents a country, and the color on the heat map changes gradually from cool to warm, indicating low to high cooperation.

**Figure 5.**
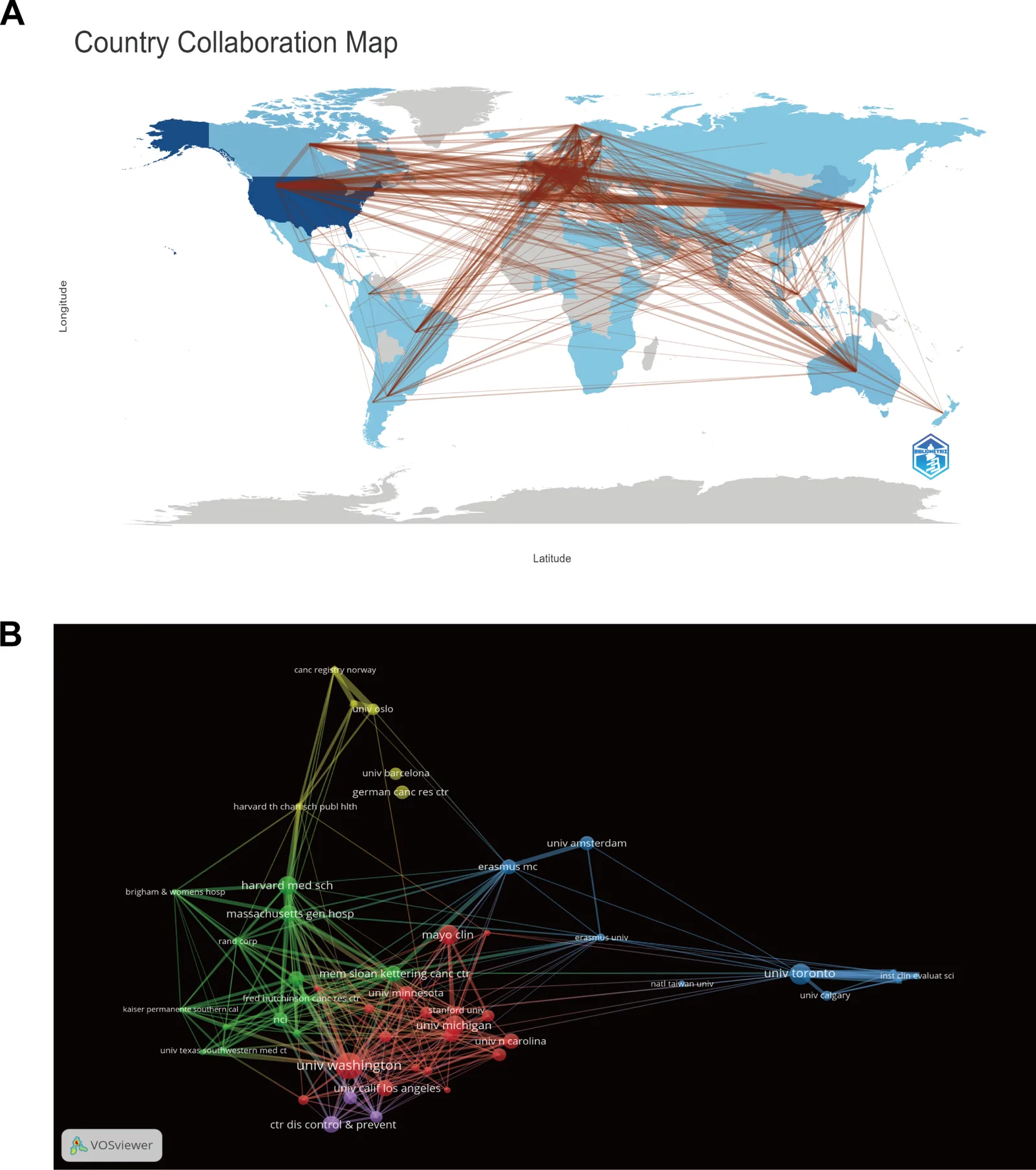
Visualization of country and institution collaboration. (A) Shows the cooperative relationships between different countries. Lines are used to connect the countries, representing their collaboration. The thickness and color intensity of the lines represent the strength or frequency of cooperation, with darker colors and thicker lines indicating closer collaboration. (B) Shows the cooperative relationships between different institutions; the nodes represent institutions, and the size of the nodes is related to the institution’s research output or number of collaborations. The lines between nodes indicate cooperative relationships.

### 3.3. Author and co-cited author analysis

Through author analysis, core researchers with significant academic influence in this field can be identified. The results indicate that a total of 14,770 authors have contributed to research in this area. The top 10 authors by publication volume are listed in Table 3. Currently, Lansdorp-Vogelaar I leads with 95 publications, an H-index of 32, a total of 3293 citations, and 1075 local citations, demonstrating outstanding academic influence. Dekker E follows closely with 73 publications, an H-index of 25, and a total of 1794 citations. Further analysis reveals that these 2 authors not only exhibit high productivity but also maintain extensive collaborative networks (Figs. 6A–B), reflecting active academic cooperation within the field of CRC screening. Although Zauber AG ranks sixth in total publication volume, the author ranks first in average citations per paper, indicating the high authority of her publications. Supplementary Figure 1, Supplemental Digital Content 2 presents a co-citation network map of the major authors. In this map, Rex, DK, and Breithauer, H are represented by large nodes with dense connections, suggesting that their research findings are frequently co-cited, underscoring their significant academic standing in the field. In summary, this study identifies a core group of authors in the field of CRC screening who play a pivotal role in advancing the discipline and facilitating academic exchange and knowledge dissemination.

**Table 3:** Top 10 most productive authors ranked by number of articles.

Rank	Authors	Articles	H index	LC	TC	TC/Articles
1	LANSDORP-VOGELAAR I	95	32	1075	3293	34.7
2	DEKKER E	73	25	519	1794	24.6
3	BRENNER H	48	21	349	1473	30.7
4	SPAANDER MCW	48	20	343	1135	23.6
5	CORONADO GD	43	15	303	793	18.4
6	ZAUBER AG	43	26	932	3050	70.9
7	GUPTA S	40	19	526	1588	39.7
8	HASSAN C	40	22	270	1359	34
9	SENORE C	39	23	467	1883	48.3
10	VON WAGNER C	37	19	298	979	26.5

LC = local citations, TC = total citations.

**Figure 6.**
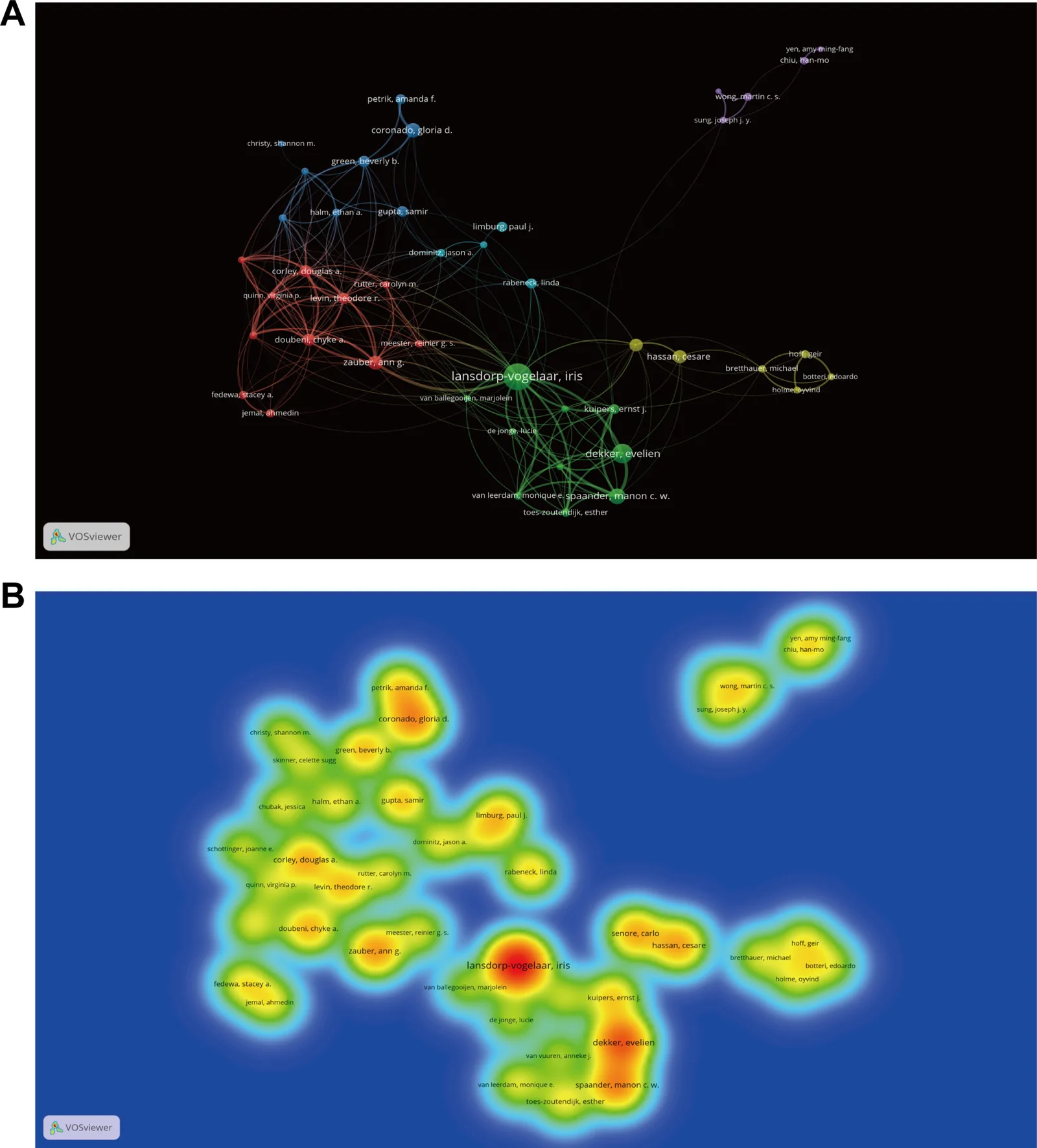
The visualization of cooperation among authors by VOS viewer. (A) Diagram of the collaborative network among authors. The same-color logo indicates the author cluster group, the thickness of the line reflects the closeness of cooperation, and the node’s size corresponds to the number of papers published by the author. (B) Heat map analysis of collaboration among authors. Each label represents an author, and the colors on the heat map are graded from cool to warm, indicating a low to a high degree of cooperation.

### 3.4. Journal analysis

Bibliometric analysis of journals allowed us to identify academic journals that play a key role in driving the field of CRC screening research. A total of 578 academic journals published papers in this field. PREVENTIVE MEDICINE was the journal that published the most papers in this field, with a total of 88 papers and 1492 citations to this literature (Table 4). It is closely followed by CANCER with 68 papers published and a total of 2111 citations. There are other important journals such as CANCER EPIDEMIOLOGY BIOMARKERS & PREVENTION, JOURNAL OF MEDICAL SCREENING, and CLINICAL GASTROENTEROLOGY AND HEPATOLOGY, which also have a high publication number and citation index. The number of papers published by these journals in the field has been steadily increasing year after year (Fig. 7A). The journals with the highest number of citations out of 3245 papers in the field are GASTROENTEROLOGY, GUT, and AM J GASTROENTEROL (Supplementary Table 2, Supplemental Digital Content 3). These data suggest that they provide a channel for researchers to publish high-impact research results. The Bradford’s Law distribution plot shows that a small number of journals accounted for a large proportion of publications in this field. Based on Bradford’s Law, journals were divided into 3 zones (core, middle, and outer) according to publication counts, with each zone producing roughly 1-third of the total publications. The core zone consisted of 21 journals, including PREVENTIVE MEDICINE, CANCER, and CANCER EPIDEMIOLOGY BIOMARKERS & PREVENTION, which together contributed 6.9% of all publications (Fig. 7B). The number of journals increased progressively from the core zone to the subsequent zones, consistent with the typical Bradford distribution pattern. Next, by constructing a co-citation network graph of journals, we can help researchers identify which journals are at the core of a particular research area and how the relevance of different journals affects the structure of scholarly communication (Supplementary Figure 2, Supplemental Digital Content 4). We found that the nodes of “gastroenterology”; “GUT” occupy a large area and are closely linked to other journals, indicating that these journals are frequently co-cited in the literature in this field. The above results reveal the central role and interrelationship of related journals in the field of CRC screening research.

**Table 4:** The top10 productive journals.

Rank	Journal	Articles	H index	TC	IF (2025)	CiteScore	SJR	JCR
1	PREVENTIVE MEDICINE	88	22	1492	3.2	8	1.793	Q1
2	CANCER	68	25	2111	5.6	10.6	2.427	Q1
3	CANCER EPIDEMIOLOGY BIOMARKERS & PREVENTION	68	23	1623	3.7	6.4	1.939	Q1
4	JOURNAL OF MEDICAL SCREENING	63	16	871	2.3	5.7	0.84	Q2
5	DIGESTIVE DISEASES AND SCIENCES	60	18	1234	2.8	5.8	1.004	Q2
6	PLOS ONE	57	14	594	2.8	5.4	0.803	Q2
7	AMERICAN JOURNAL OF PREVENTIVE MEDICINE	56	18	1792	4.7	8.6	2.044	Q1
8	CLINICAL GASTROENTEROLOGY AND HEPATOLOGY	56	26	1888	16.2	21.7	3.843	Q1
9	PREVENTIVE MEDICINE REPORTS	52	11	477	2.8	4.7	1.008	Q2
10	JOURNAL OF CANCER EDUCATION	51	13	514	1.6	3.5	0.646	Q3

IF = Journal Impact Factor, JCR = Journal Citation Reports, SJR = SCImago Journal Rank, TC = Total Citations.

**Figure 7.**
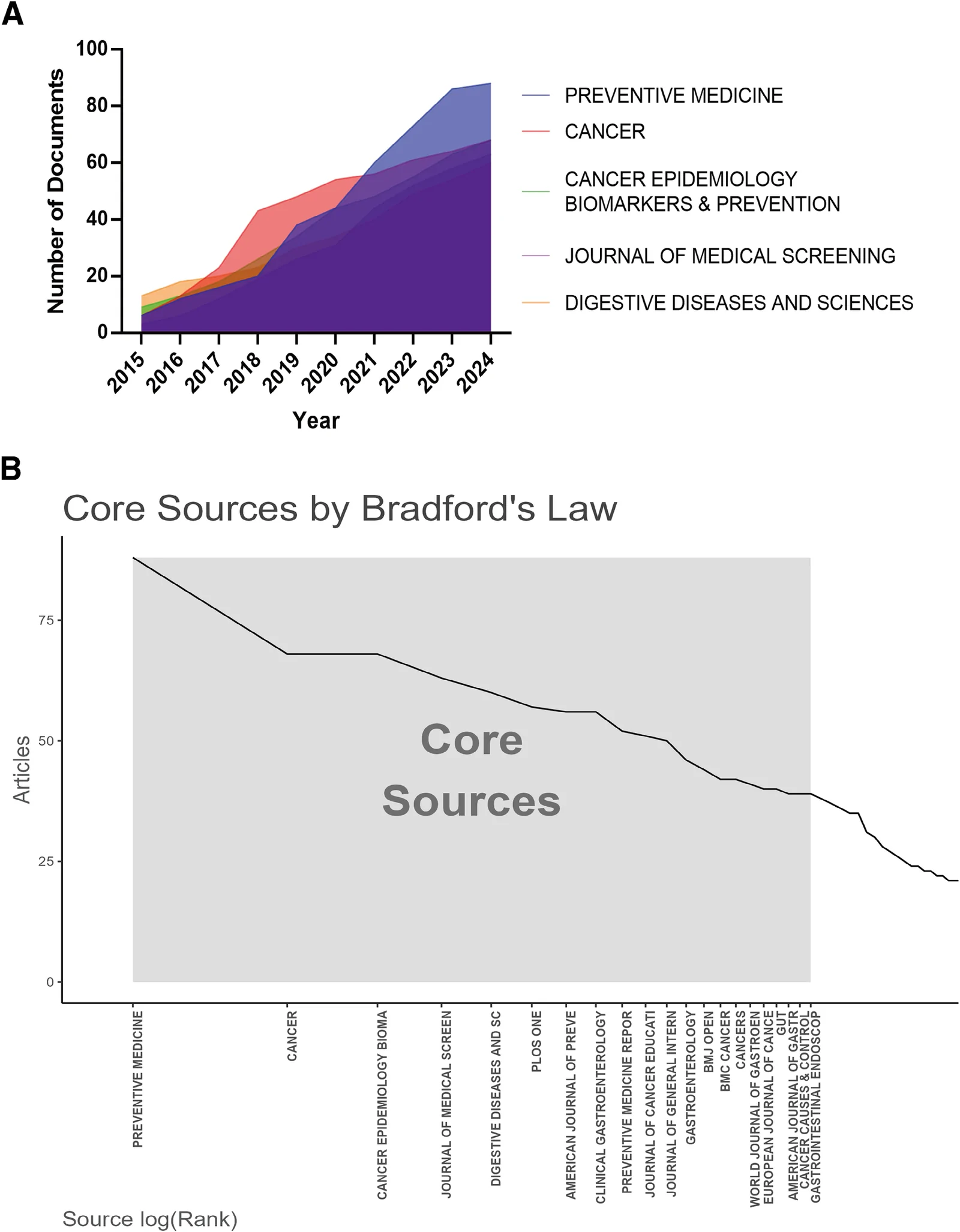
Journal publication trend and distribution map. (A) Trend chart of annual publication volume of journals. (B) Distribution map of Bradford’s Law for journals.

### 3.5. Analysis of cited papers and co-cited references

The bibliometric analysis of papers allows us to identify the core papers that have had a significant impact in the field of CRC screening research. There are 3245 papers and 61,424 citations in this field from 2015 to 2024. We showed the top 10 most influential papers in this research field, ranked based on their number of global citations (Table 5). It should be noted that the third-ranked paper, which focuses on irritable bowel syndrome, does not directly address CRC screening. However, its high citation count reflects broad clinical interest in irritable bowel syndrome, as this condition shares overlapping symptoms with CRC and is often discussed in the context of screening indications. Additionally, the coupling analysis of the papers in this field shows that nodes such as “wolf (2018)” and “schreuders (2015)” occupy a large area in the 2 figures and are closely connected to other nodes (Fig. 8), indicating that these 2 papers have high academic influence and centrality in this research field. In the co-citation network analysis graph of references, we notice that the 2 citations “bibbins-domingo k” and “wolf amd” have large node sizes and are closely linked to many other papers (Supplementary Figure 3, Supplemental Digital Content 5), indicating that these 2 papers occupy a prominent position in the co-citation analysis of the field, suggesting that they may play a central role in advancing scientific discussion and knowledge in the field. We also demonstrated the 10 most influential references in the field of this research and how they were ranked by the number of times they were cited by these 3245 papers (Supplementary Table 3, Supplemental Digital Content 6). These highly cited citations are likely to represent the core findings and trends in this research area. These important citations may have served as the basis for subsequent studies, playing an important role in understanding trends in the field, developing new therapeutic strategies, and advancing knowledge in related biomedical research areas.

**Table 5:** The top 10 global cited documents in the field.

Rank	Title	DOI	Journal	TC
1	Colorectal cancer screening for average-risk adults: 2018 guideline update from the American Cancer Society	10.3322/caac.21,457	CA CANCER J CLIN	1336
2	Colorectal cancer screening: a global overview of existing programmes	10.1136/gutjnl-2014–309086	GUT	929
3	Irritable bowel syndrome: a clinical review	10.1001/jama.2015.0954	J AM MED ASSOC	753
4	Worldwide burden of colorectal cancer: a review	10.1007/s13304–016-0359-y	UPDATES SURG	547
5	Colorectal Cancer Screening: Recommendations for Physicians and Patients from the U.S. Multi-Society Task Force on Colorectal Cancer	10.1038/ajg.2017.174	AM J GASTROENTEROL	468
6	ACG Clinical Guidelines: Colorectal Cancer Screening 2021	10.14309/ajg.0000000000001122	AM J GASTROENTEROL	422
7	Colorectal cancer screening: Recommendations for physicians and patients from the U.S. Multi-Society Task Force on Colorectal Cancer	10.1016/j.gie.2017.04.003	GASTROINTEST ENDOSC	418
8	Cancer screening in the United States, 2018: A review of current American Cancer Society guidelines and current issues in cancer screening	10.3322/caac.21,446	CA CANCER J CLIN	414
9	Colorectal cancer population screening programs worldwide in 2016: An update	10.3748/wjg.v23.i20.3632	WORLD J GASTROENTERO	412
10	Colorectal cancer screening: An updated review of the available options	10.3748/wjg.v23.i28.5086	WORLD J GASTROENTERO	400

TC = total citations.

**Figure 8.**
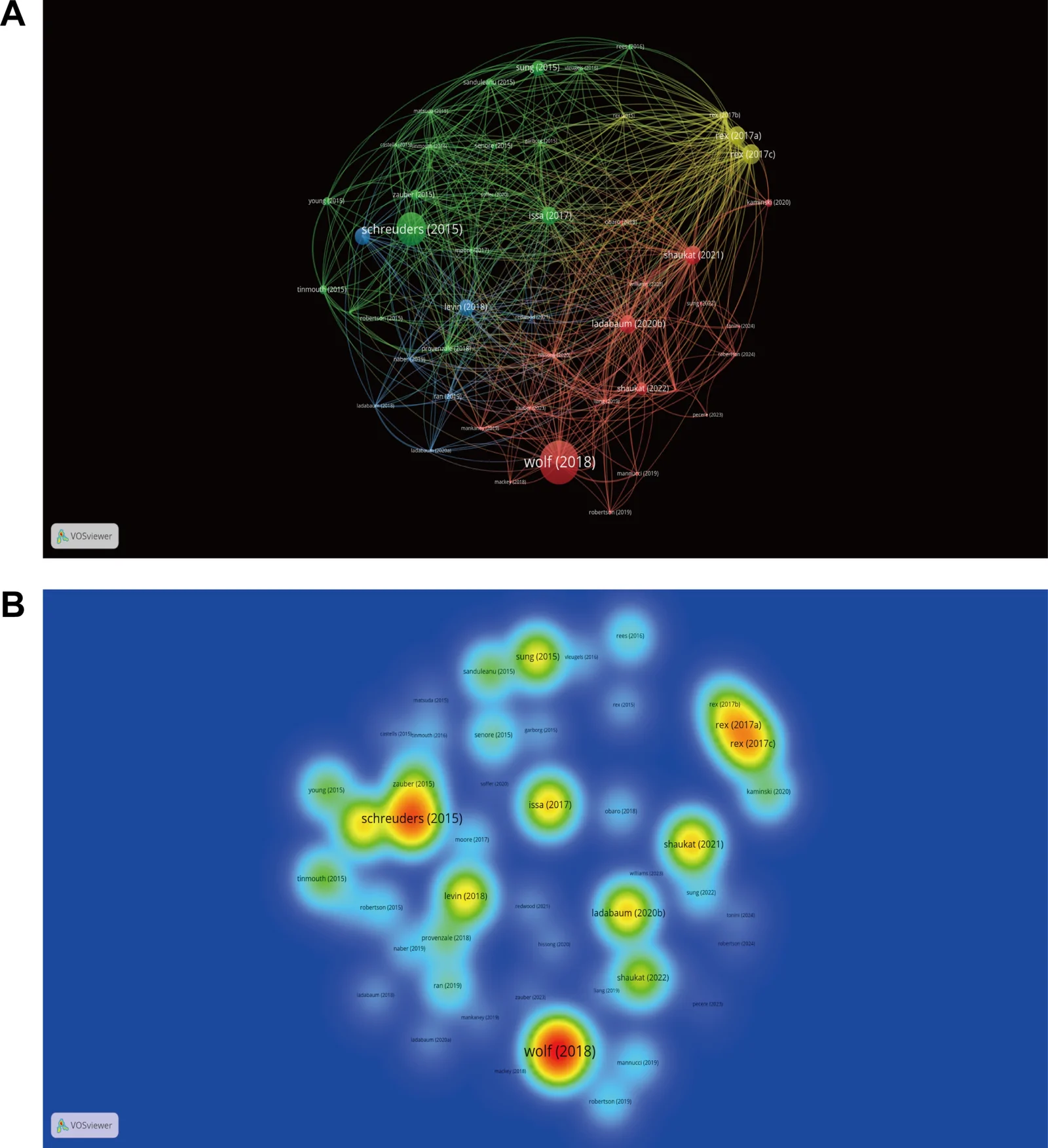
Coupling analysis of articles by VOS viewer. (A) Coupling analysis of related research outcomes. Each node represents an independent research outcome, and the size of the node reflects the number of citations contributed by the research outcome in the coupling analysis. The larger the node, the higher the coupling degree of the research outcome with other research outcomes. The lines indicate coupling relationships between research outcomes, and the same color indicates the same cluster group of research outcomes. (B) Heatmap analysis of coupling in related research outcomes. Each label represents an independent research outcome, and the colors on the heatmap gradually shift from cool to warm, marking the coupling degree of independent research outcomes from low to high.

### 3.6. Author keywords analysis

Keywords are a fundamental part of research papers. High-frequency keywords reflect the main research focus during a specific period and indicate research hotspots and emerging areas.^[16]^ In this study, 4229 author keywords were included in this analysis. We displayed the 20 author keywords with the highest frequency, with “colonoscopy” ranking first at 436 occurrences, suggesting an in-depth study of colonoscopy and its effective application by researchers in the field (Table 6). Other CRC screening methods include “fecal immunochemical test (FIT),” “fecal occult blood test.” In terms of prevention, the main keywords include “cancer prevention,” “prevention,” and “primary care” (Figs. 9A–B). The keywords indicate that early screening plays a vital role in CRC prevention. In the upper right corner of the Theme Development Chart, the keywords “colonoscopy,” “FIT,” and “fecal occult blood test” showed high relevance to the theme and a high degree of development. In the lower right corner, the presence of “early detection of cancer,” “mass screening,” and “cancer prevention” indicate that these themes are highly relevant but less developed (Fig. 9C). At the same time, these 3 domains represent core research themes, indicating that CRC screening plays an important role in tumor prevention. Keyword cluster analysis was performed using CiteSpace software with the log-likelihood ratio algorithm. The cluster map contained 266 nodes and 1080 links, with a network density of 0.0306. The Q value was 0.4166 and the *S* value was 0.7243, indicating good clustering quality and high reliability (Fig. 10C). A total of 7 clusters were formed, including cancer screening, FIT, risk, knowledge, adenoma detection rate, CRC, biomarkers, and preferences. The main content under each cluster label is listed in Supplementary Table 4, Supplemental Digital Content 7. In addition, we showed the author keyword co‑occurrence network, with larger nodes representing higher frequency (Fig. 10A). Burst keyword analysis further revealed shifting research priorities. “Artificial intelligence (AI)” had the strongest burst from 2022 to 2024, “Fecal occult blood test” had a strong burst from 2017 to 2020, “Health literacy” burst from 2019 to 2021. Early bursts included “interval cancer,” “flexible sigmoidoscopy,” and “colorectal adenoma.” Recent bursts included “screening colonoscopy” and “risk factors” (Fig. 10B). These findings indicate a shift in research focus from traditional screening methods to emerging technologies and patient-related factors.

**Table 6:** Top 20 Author Keywords with the most frequency in colorectal cancer screening.

Rank	Words	Frequency
1	Colonoscopy	436
2	Fecal immunochemical test	187
3	Early detection of cancer	125
4	Mass screening	95
5	Fecal occult blood test	91
6	Cancer	74
7	Cancer prevention	64
8	Prevention	54
9	Primary care	53
10	Adenoma	50
11	Covid-19	49
12	Early detection	47
13	Breast cancer	46
14	Public health	45
15	Endoscopy	44
16	Epidemiology	43
17	FIT	43
18	Participation	40
19	Cost-effectiveness	39
20	Adenoma detection rate	38

**Figure 9.**
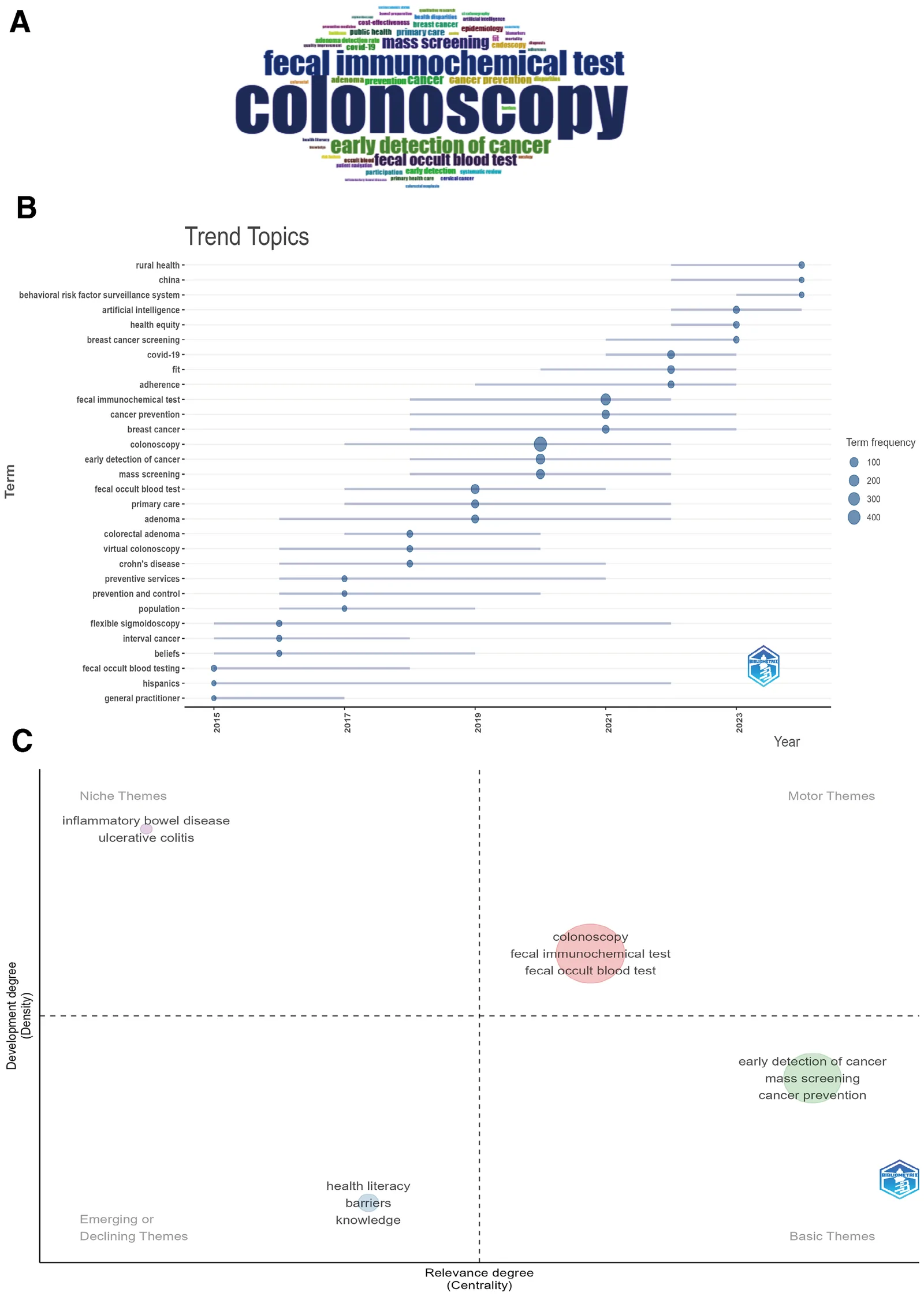
Author keyword analysis using the R-based bibliometrix package. (A) The word cloud shows the author keywords in the field. The size of the author keywords is proportional to their frequency of occurrence; the larger the word, the more frequently it appears in the relevant literature. (B) Trend topics show the active periods of the commonly used author keywords. (C) Topic development and correlation analysis diagram, used to display the development and correlation of different topics within the field. The horizontal axis represents the degree of correlation, and the vertical axis represents the degree of development.

**Figure 10.**
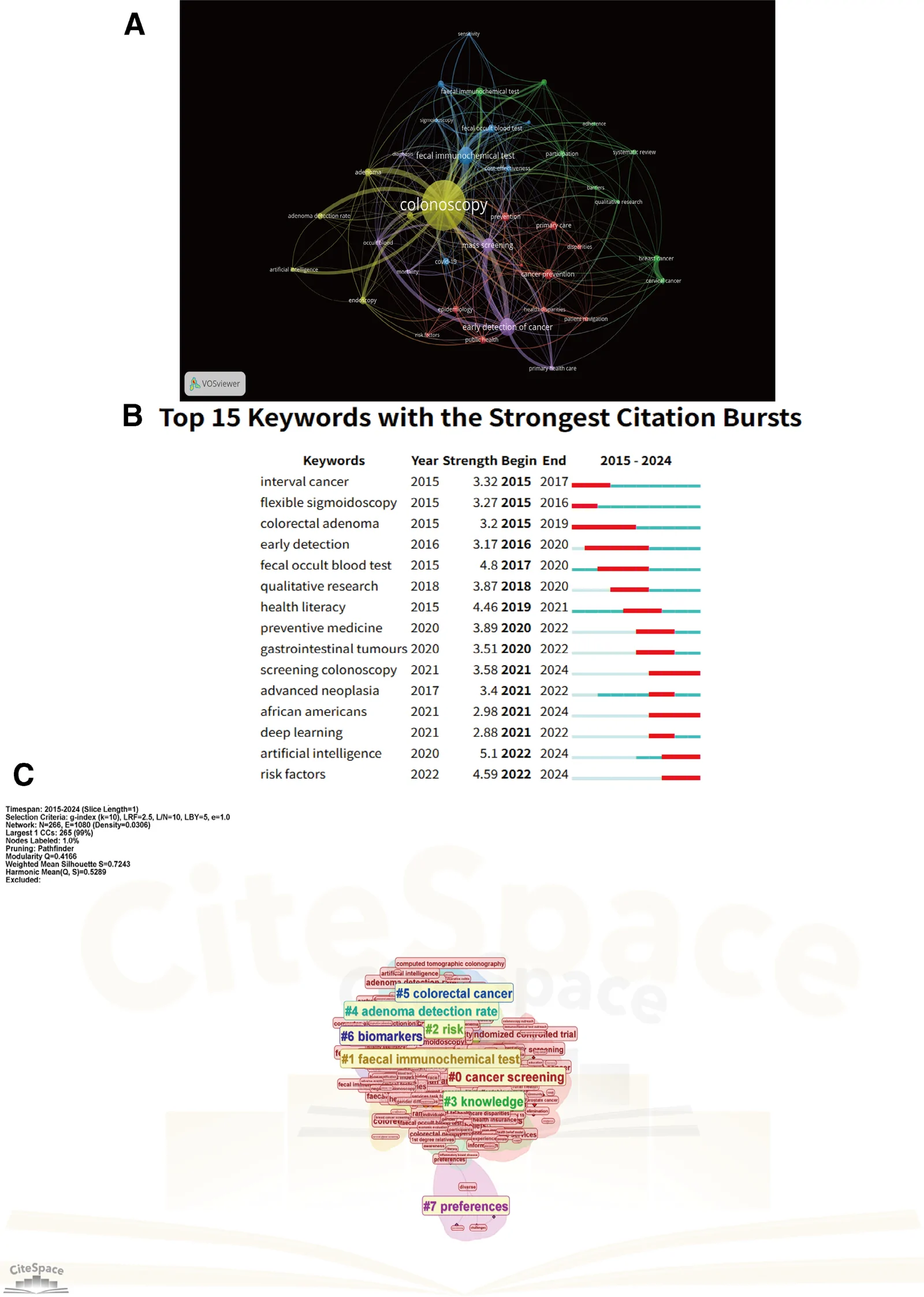
Author keywords analysis by VOS viewer and CiteSpace. (A) Author keywords co-occurrence analysis. Each node represents an author keyword, and the node size reflects the occurrence frequency of the author keyword. The larger the node, the more frequent the occurrence of the author keyword. The line indicates the co-occurrence relationship between the author keywords, and the same color represents the same author keywords cluster group. (B) Top 15 keywords with the strongest citation bursts. The red segments indicate the burst periods, showing the start year, end year, and duration of each burst. (C) Keyword co-occurrence network of CRC screening research based on CiteSpace. Each node represents a keyword, with node size reflecting its frequency. Different colors indicate distinct clusters, labeled by representative terms.

## 4. Discussion

### 4.1. International Trends in CRC Screening Research

Given the poor prognosis of advanced CRC, extensive research has been conducted to find accurate and efficient methods for screening and early diagnosis. In this study, we performed a bibliometric analysis to evaluate publications in the field of CRC screening. A total of 3245 publications from 2015 to 2024 were identified from the Web of Science Core Collection.

The annual number of publications reflects, to a certain extent, the interest in this field. Our results showed that the number of publications grew steadily from 2015 to 2019 and increased more rapidly after 2020. The United States ranked first in both publication volume and total citations, followed by China and the Netherlands. Additionally, the University of North Carolina ranked first in publication output among all institutions. The United States’ leading position can be attributed to sustained funding, established screening programs, and strong research infrastructure.^[17,18]^ The Netherlands had the highest average citations per paper and international collaboration rate, reflecting its integrated screening program and collaborative research culture.^[19,20]^ China ranked second in publication volume due to rapid growth in government research funding and its large population base for clinical research. However, its citation impact remains low, likely due to the recent expansion of research output. In contrast, most low- and middle-income countries, including Egypt, India, and Lebanon, are underrepresented in CRC screening research. This is not because CRC is not a concern in these regions. Large-scale screening studies require infrastructure, registries, and funding, which are often lacking in these countries.^[21]^ Research opportunities and high-impact publications also tend to favor high-income countries. This imbalance raises concerns about whether current screening evidence and AI-assisted tools can be generalized to diverse populations worldwide.

### 4.2. Colonoscopy screening: a core focus of international research

CRC ranks among the world’s most prevalent malignancies, and early screening is crucial for reducing its burden.^[22]^ Keyword co‑occurrence analysis identified “colonoscopy” as the most frequent keyword among 4242 author keywords, confirming its central role in CRC screening research. Although numerous screening methods exist, colonoscopy remains the recognized gold standard.^[23]^ It is the only screening method capable of both detecting tumors and enabling early prevention through the removal of precancerous lesions, offering the advantages of high sensitivity and specificity. Flexible sigmoidoscopy in individuals aged 55 to 64 reduces the cumulative incidence of CRC by 24% and the cumulative mortality rate by 25%.^[24]^ Thomas Heisser et al also found that colonoscopy enables early detection of lesions, significantly reducing CRC mortality rates.^[25]^ The keyword trend chart further showed that colonoscopy‑related research peaked in 2020, reflecting sustained research interest over the study period.

The quality of colonoscopy may be influenced by factors such as population screening participation and patient compliance. High-quality colonoscopy has been shown to reduce CRC incidence more effectively than low-quality colonoscopy.^[26]^ Its quality depends on patient compliance, bowel preparation,^[5]^ endoscopic manipulation techniques such as cecal intubation rate,^[27]^ and physician‑related factors including adenoma detection rate and withdrawal time.^[28]^ Therefore, maintaining high quality is essential for effective screening. Concurrent use of FIT and colonoscopy screening can improve patient survival rates^[29]^ and adenoma detection rates.^[30]^ Notably, keyword burst analysis showed that “screening colonoscopy” remained a burst keyword from 2021 to 2024, indicating that this topic continues to evolve with technological advances. With the development of AI, endoscopy-assisted technologies such as endoscopic ultrasound, electronic endoscopy, and computer-aided diagnosis have been applied clinically, improving detection efficiency and accuracy.^[31]^ However, colonoscopy is invasive and costly. Future efforts should address issues such as interval cancers, patient discomfort, bowel injury, and optimal screening intervals for high-risk populations.

### 4.3. Secondary hotspots in CRC screening research

FIT plays a prominent role in CRC screening research. Our analysis showed that it ranked as the second most frequent keyword. As a noninvasive screening method, it offers advantages such as simple operation and low cost,^[32]^ and is one of the most widely used approaches for early CRC screening. Compared to traditional fecal occult blood tests, FIT offers higher specificity and sensitivity.^[4]^ Trend Topics analysis further showed that “FIT” was a consistent research theme from 2018 to 2022, peaking in 2021, indicating that FIT received extensive and sustained research attention during this period. In the keyword co-occurrence network, “FIT” served as the leading node of a dedicated cluster (Cluster 1), alongside other screening-related terms such as “cancer screening” (Cluster 0) and “risk” (Cluster 2), highlighting its central position in the field’s knowledge structure. A 15-year study confirmed that FIT screening reduces both the incidence and mortality of CRC.^[33]^ Additionally, Zhang et al demonstrated through a meta-analysis that FIT screening can reduce the incidence of CRC by 21% and lower CRC mortality by 59%.^[34]^ Currently, annual or biennial FIT is cost-effective^[35]^ and linked to fewer CRC deaths, especially for left-sided tumors.^[36]^ To enhance testing efficiency, Niedermaier et al found that lowering the FIT positive threshold improves detection sensitivity, opening new avenues for the application of FIT.^[37]^ In addition, under all threshold conditions, the FIT demonstrated superior cost-effectiveness compared to gFOBT.^[38]^ The cost-effectiveness advantage depends on adequate screening uptake and follow-up adherence, which are closely linked to patient health literacy, a topic that showed a strong citation burst from 2019 to 2021. However, the FIT demonstrates lower diagnostic accuracy in high-risk populations compared to those at average risk^[39]^ and positive results still require confirmation by colonoscopy. This limitation suggests that FIT and colonoscopy are not substitutes but rather complementary components of a stratified screening strategy.

### 4.4. Emerging areas for sustainable focus in CRC screening research

All keywords were grouped into 7 clusters, including cancer screening, FIT, risk, knowledge, adenoma detection rate, CRC, biomarkers, and preferences. Based on the average start year of each cluster, the analysis was divided into 2 periods. The first period was before 2019. During this period, the annual publication output grew slowly. Research mainly focused on CRC screening populations and screening methods, including CT colonography,^[40]^ FIT,^[41]^ woman, and adenoma detection. Analytical techniques were limited, and screening coverage remained narrow. The second period was after 2019. During this period, the annual publication output increased rapidly, and developing early diagnostic biomarkers became a major research direction.

Currently, biomarkers are mainly divided into serum biomarkers and fecal biomarkers. Among serum-based markers, circulating tumor cells and circulating tumor DNA are the most widely studied.^[42]^ Both are noninvasive, convenient, and show high sensitivity and specificity for tumor detection.^[43]^ Studies have shown that CRC patients with positive circulating tumor cells have shorter progression-free survival and overall survival,^[44]^ as well as higher recurrence rates.^[45]^ Circulating tumor cells, as a nearly noninvasive detection method, will play an increasingly vital role in tumor diagnosis, detection, and prognosis in the future. Circulating tumor DNA (ctDNA) is a form of cell-free DNA in the blood, but its concentration is relatively low, accounting for only 1%.^[46]^ Cell-free DNA testing shows a sensitivity of 87.5% for stage I-III CRC and a specificity of 89.6% for advanced tumors.^[47]^ Compared with traditional biopsies, ctDNA is noninvasive and can reflect tumor heterogeneity.^[48]^ It also has a short half-life, making it suitable for real-time monitoring. However, its detection may be limited by the small amount of DNA released from early-stage tumors. Bessa et al developed a multimodal ctDNA-based blood test integrating genomics, epigenomics, fragmentomics, and proteomics, achieving 93% sensitivity and 90% specificity for CRC detection, with up to 96% sensitivity in stage III disease.^[49]^ In clinical practice, mutations in target genes such as SEPT9 are often detected in ctDNA. SEPT9 methylation testing has shown 90% sensitivity and 88% specificity for early-stage CRC and has been approved for early diagnosis.^[50,51]^

For fecal biomarkers, fecal DNA testing is a widely used technique that detects genetic mutations and methylation in shed colorectal cells. A next-generation multi-target fecal DNA test showed 93.9% sensitivity for CRC and 90.6% specificity for advanced lesions, superior to FIT for both cancer and advanced precancerous lesions.^[52]^ Given colorectal tumor heterogeneity, a single method has limited sensitivity. FIT combined with fecal DNA testing achieved 91.9% sensitivity for CRC, compared to 62.4% for FIT alone.^[53]^ Combining FIT with fecal DNA testing can improve early detection. However, this approach remains impractical for large-scale screening due to high costs and self-collection of stool samples. The tumor microbiome demonstrates significant potential as a novel biomarker in CRC screening.^[54]^ High-throughput sequencing reveals strong associations between microbial profiles and CRC.^[55]^ Dysbiosis of gut fungi and bacteria has been linked to CRC development and progression,^[56,57]^ and certain microbial markers have also shown potential for early detection.^[58]^ In our co-occurrence network, microbiome-related topics were grouped into biomarkers (Cluster 6), suggesting that they may become a research focus in CRC screening.

Our keyword burst analysis revealed that “AI,” “fecal occult blood test,” “health literacy,” and “risk factors” were the main research hotspots in CRC screening. In addition, “qualitative research,” “screening colonoscopy,” and “african americans” also showed strong bursts. Regarding future trends in CRC screening research, first, the bursts of “health literacy” and “qualitative research” reflect sustained research interest in patient cognition and barriers to screening. Improving screening adherence remains an important direction. Second, the burst of “african americans” suggests that disparities in screening across different populations and regions have become a research priority. This requires attention to differences in economic development and healthcare resource allocation across regions. Additionally, future studies should compare different screening methods, strategies, and age cutoffs to identify the most cost-effective approaches. Third, the bursts of “AI” and “deep learning” indicate that AI applications in CRC screening are becoming the most active research frontier.^[59]^ AI can rapidly identify relevant information from complex data, reduce errors, and improve efficiency. Current AI applications in CRC screening mainly focus on computer-aided detection and diagnosis.^[60,61]^ AI-assisted digestive endoscopy products have been gradually introduced into clinical practice, which may help improve adenoma and polyp detection rates in clinical settings.^[59,62]^

## 5. Strengths and limitations

This study presents a comprehensive bibliometric analysis of CRC screening research, highlighting key contributors, major research themes, and evolving frontiers to help researchers navigate the field. This study also has several limitations. First, our analysis relied solely on the Web of Science Core Collection, which predominantly indexes English-language journals and may underrepresent research published in other languages or regional journals. Second, citation-based metrics are subject to citation-age effects, which may inflate the impact of early publications while underestimating recent high-quality studies. Third, self-citation practices and regional differences in publication practices (e.g., journal accessibility, language barriers) may influence author and institutional rankings. Fourth, the use of author-defined keywords may introduce variability across publications, as different authors may use different terms for the same concept. Fifth, the underrepresentation of countries with smaller populations or lower research output does not necessarily reflect the quality of their research but rather the inherent characteristics of bibliometric data aggregation. These limitations should be considered when interpreting the findings, and future studies could address these issues by incorporating multiple databases and using normalized impact indicators.

## 6. Conclusion

This study employs bibliometric methodologies to evaluate the research landscape of CRC screening technologies from 2015 to 2024, analyzing literature across various dimensions including publication year, country, institution, author, journal, and keyword. The findings indicate a sustained increase in scholarly attention to CRC screening, peaking in 2022, underscoring its growing strategic significance in global public health initiatives. The technological evolution in this field is moving from traditional screening tools, such as colonoscopy and FIT, toward emerging technologies like AI‑assisted detection. This progression reflects a paradigm shift toward more intelligent, and personalized screening approaches, signaling a transition toward precision prevention and efficient clinical intervention.

Presently, CRC screening technologies are undergoing rapid development. Global research collaboration and innovation are pivotal to enhancing their accessibility and effectiveness. By reinforcing fundamental research, advancing supportive health policies, and fostering international cooperation, the goals of early detection, diagnosis, and treatment of CRC can be achieved.

## Acknowledgments

This work was supported by the Nanchong Municipal-University Science and Technology Strategic Cooperation Project (Grant No. 22SXQT0077), the University-level Scientific Research Development Fund of North Sichuan Medical College (Grant No. CBY22-QNA12), the Scientific Research Development Project of the Affiliated Hospital of North Sichuan Medical College (Grant Nos. 2023PTZK008 and 2024GC004), and the Guang’an Science and Technology Innovation Project (Grant No. 2024SYF17). The funders had no role in the study design, data collection, analysis, interpretation, or manuscript preparation. The authors declare no conflicts of interest. The authors would like to thank the participating centers and members.

## Supplementary Material

The original contributions presented in the study are included in the article/Supplementary Material. Further inquiries can be directed to the corresponding authors.

## Author contributions

**Conceptualization:** Jinkai Zhou, He Zhou, Jiayu Xu, Maohua Wang, Lei Liu, Xiaolong Tang.

**Data curation:** Jinkai Zhou, He Zhou, Jiayu Xu, Maohua Wang, Lei Liu, Xiaolong Tang.

**Formal analysis:** Jinkai Zhou, Maohua Wang, Lei Liu, Xiaolong Tang.

**Writing – original draft:** Jinkai Zhou, He Zhou, Jiayu Xu, Maohua Wang, Lei Liu.














