# Supplementary material for: Research trends and hotspots in colorectal cancer screening: Bibliometric and visualization analysis (2015–2024)
Source: Medicine (Baltimore). 2026 Oct 16;105(42):e51198. doi: 10.1097/MD.0000000000051198 (PMC13681169; doi:10.1097/MD.0000000000051198)
Supplement: Supplementary file 1 [file medi-105-e51198-s001.docx]

**Supplementary Table1**. The top 8 productive institutions

| **Rank** | **Affiliation** | **Country** | **Articles** |
| --- | --- | --- | --- |
| 1 | UNIVERSITY OF NORTH CAROLINA (including Chapel Hill) | United States | 488 |
| 2 | ERASMUS UNIVERSITY ROTTERDAM (including Erasmus MC) | Netherlands | 483 |
| 3 | UNIVERSITY OF CALIFORNIA SYSTEM | United States | 397 |
| 4 | HARVARD UNIVERSITY | United States | 390 |
| 5 | UNIVERSITY OF TORONTO | Canada | 304 |
| 6 | KAISER PERMANENTE | United States | 262 |
| 7 | UNIVERSITY OF TEXAS SYSTEM | United States | 261 |
| 8 | HARVARD UNIVERSITY MEDICAL AFFILIATES | United States | 241 |

“University of North Carolina” and “University of North Carolina Chapel Hill” were merged as “University of North Carolina”; “Erasmus University Rotterdam” and “Erasmus MC” were merged as “Erasmus University Rotterdam”
