# Supplementary material for: Research trends and hotspots in colorectal cancer screening: Bibliometric and visualization analysis (2015–2024)
Source: Medicine (Baltimore). 2026 Oct 16;105(42):e51198. doi: 10.1097/MD.0000000000051198 (PMC13681169; doi:10.1097/MD.0000000000051198)
Supplement: Supplementary file 2 [file medi-105-e51198-s003.docx]

**Supplementary Table 2**. Top 10 most cited journals and their local citation counts

| **Rank** | **Journal** | **Citation count** |
| --- | --- | --- |
| 1 | GASTROENTEROLOGY | 4821 |
| 2 | GUT | 3885 |
| 3 | AM J GASTROENTEROL | 3596 |
| 4 | NEW ENGL J MED | 3558 |
| 5 | GASTROINTEST ENDOSC | 3071 |
| 6 | ANN INTERN MED | 2830 |
| 7 | JAMA-J AM MED ASSOC | 2583 |
| 8 | CANCER-AM CANCER SOC | 2460 |
| 9 | CANCER EPIDEM BIOMAR | 2416 |
| 10 | CA-CANCER J CLIN | 1993 |
