# Supplementary material for: Research trends and hotspots in colorectal cancer screening: Bibliometric and visualization analysis (2015–2024)
Source: Medicine (Baltimore). 2026 Oct 16;105(42):e51198. doi: 10.1097/MD.0000000000051198 (PMC13681169; doi:10.1097/MD.0000000000051198)
Supplement: Supplementary file 3 [file medi-105-e51198-s006.docx]

**Supplementary Table 3**. The top 10 local cited references in the field

| **Rank** | **Title** | **DOI** | **Journal** | **Citations** |
| --- | --- | --- | --- | --- |
| 1 | Screening for Colorectal Cancer: US Preventive Services Task Force Recommendation Statement | 10.1001/JAMA.2016.5989 | JAMA-J AM MED ASSOC | 397 |
| 2 | Colorectal cancer screening for average-risk adults: 2018 guideline update from the American Cancer Society | 10.3322/CAAC.21457 | CA-CANCER J CLIN | 305 |
| 3 | Colorectal cancer screening: a global overview of existing programmes | 10.1136/GUTJNL-2014-309086 | GUT | 260 |
| 4 | Multitarget stool DNA testing for colorectal-cancer screening | 10.1056/NEJMc1405215 | NEW ENGL J MED | 255 |
| 5 | Screening for Colorectal Cancer: US Preventive Services Task Force Recommendation Statement | 10.1001/JAMA.2021.6238 | JAMA-J AM MED ASSOC | 252 |
| 6 | Colonoscopic polypectomy and long-term prevention of colorectal-cancer deaths | 10.1056/NEJMOA1100370 | NEW ENGL J MED | 216 |
| 7 | Screening for colorectal cancer: U.S. Preventive Services Task Force recommendation statement | 10.7326/0003-4819-149-9-200811040-00243 | ANN INTERN MED | 207 |
| 8 | Reducing mortality from colorectal cancer by screening for fecal occult blood. Minnesota Colon Cancer Control Study | 10.1056/NEJM199305133281901 | NEW ENGL J MED | 203 |
| 9 | Randomised controlled trial of faecal-occult-blood screening for colorectal cancer | 10.1016/S0140-6736(96)03386-7 | LANCET | 199 |
| 10 | Long-term mortality after screening for colorectal cancer | 10.1056/NEJMOA1300720 | NEW ENGL J MED | 198 |
