# Supplementary material for: Research trends and hotspots in colorectal cancer screening: Bibliometric and visualization analysis (2015–2024)
Source: Medicine (Baltimore). 2026 Oct 16;105(42):e51198. doi: 10.1097/MD.0000000000051198 (PMC13681169; doi:10.1097/MD.0000000000051198)
Supplement: Supplementary file 4 [file medi-105-e51198-s007.docx]

**Supplementary Table 4.** Summary of keyword clusters.

| **ClusterID** | **Number of keywords** | **Average year** | **Cluster name** | **Major keywords** |
| --- | --- | --- | --- | --- |
| 0 | 68 | 2017 | cancer screening | cancer screening; women; united states; care; disparity |
| 1 | 40 | 2016 | faecal immunochemical test | faecal immunochemical test; colorectal neoplasms; fecal immunochemical test; colorectal cancer screening; cancer screening |
| 2 | 38 | 2017 | risk | risk; colon; polyps; risk factors; prevalence |
| 3 | 36 | 2017 | knowledge | knowledge; health literacy; decision making; faecal occult blood test; information |
| 4 | 36 | 2018 | adenoma detection rate | adenoma detection rate; artificial intelligence; society task force; polypectomy; surveillance |
| 5 | 35 | 2016 | colorectal cancer | colorectal cancer; ct colonography; mortality; virtual colonoscopy; cost effectiveness |
| 6 | 7 | 2020 | biomarkers | biomarkers; validation; informed decision making; risk assessment; benefits |
| 7 | 5 | 2018 | preferences | preferences; smartphone; case-note review; formats; informed choice |
